# Two Amnion-Derived Mesenchymal Stem-Cells Injections to Osteoarthritic Elbows in Dogs—Pilot Study

**DOI:** 10.3390/ani13132195

**Published:** 2023-07-04

**Authors:** Michal Domaniza, Marian Hluchy, Dasa Cizkova, Filip Humenik, Lucia Slovinska, Nikola Hudakova, Lubica Hornakova, Juraj Vozar, Alexandra Trbolova

**Affiliations:** 1 Small Animal Hospital, University of Veterinary Medicine and Pharmacy, Komenskeho 73, 041 81 Kosice, Slovakia; 2 Centre of Experimental and Clinical Regenerative Medicine, University of Veterinary Medicine and Pharmacy, Komenskeho 68/73, 041 81 Kosice, Slovakia; 3 Associated Tissue Bank, Faculty of Medicine, P.J. Safarik University and L.Pasteur University Hospital, Trieda SNP 1, 040 11 Kosice, Slovakia

**Keywords:** osteoarthritis, dog, mesenchymal stem cells, regenerative medicine

## Abstract

**Simple Summary:**

Osteoarthritis is a degenerative disease of synovial joints characterized by joint inflammation, damage of articular cartilage, and degenerative changes in periarticular tissues. The onset of lameness, pain, and joint stiffness are usually the first clinical signs observed by the owner. In recent years, many conventional therapies (NSAIDs, opioids, paracetamol, and gabapentinoids) for osteoarthritis have been described. Due to the complex mechanism of pain, multimodal analgesia in OA treatment is necessary. Unfortunately, conventional therapy does not offer the regenerative abilities of damaged tissue. However, mesenchymal stem cells have a definite advantage in chondrogenic, immunomodulatory, anti-apoptotic, and anti-fibrotic effects in joint environments in contrast to conventional therapies.

**Abstract:**

The aim of the study was to investigate the potential of cell-based regenerative therapy for elbow joints affected by osteoarthritis. Interest was focused on two intra-articular applications of amnion-derived mesenchymal stem cells (A-MSCs) to a group of different breeds of dogs with elbow osteoarthritis (13 joints). Two injections were performed 14 days apart. We evaluated synovial fluid biomarkers, such as IFN-γ, IL-6, IL-15, IL-10, MCP-1, TNF-α, and GM-CSF, by multiplex fluorescent micro-bead immunoassay in the treated group of elbows (n = 13) (day 0, day 14, and day 28) and in the control group of elbows (n = 9). Kinematic gait analysis determined the joint range of motion (ROM) before and after each A-MSCs application. Kinematic gait analysis was performed on day 0, day 14, and day 28. Kinematic gait analysis pointed out improvement in the average range of motion of elbow joints from day 0 (38.45 ± 5.74°), day 14 (41.7 ± 6.04°), and day 28 (44.78 ± 4.69°) with statistical significance (*p* < 0.05) in nine elbows. Correlation analyses proved statistical significance (*p* < 0.05) in associations between ROM (day 0, day 14, and day 28) and IFN-γ, IL-6, IL-15, MCP-1, TNF-α, and GM-CSF concentrations (day 0, day 14, and day 28). IFN-γ, IL-6, IL-15, MCP-1, GM-CSF, and TNF- α showed negative correlation with ROM at day 0, day 14, and day 28, while IL-10 demonstrated positive correlation with ROM. As a consequence of A-MSC application to the elbow joint, we detected a statistically significant (*p* < 0.05) decrease in concentration levels between day 0 and day 28 for IFN-γ, IL-6, and TNF-α and statistically significant increase for IL-10. Statistical significance (*p* < 0.05) was detected in TNF-α, IFN-γ, and GM-CSF concentrations between day 14 and the control group as well as at day 28 and the control group. IL-6 concentrations showed statistical significance (*p* < 0.05) between day 14 and the control group.

## 1. Introduction

Osteoarthritis (OA) is an inflammatory condition of synovial joints (SJ) which manifests with clinical signs such as lameness, pain, and decreased range of motion. Any joint can be affected by OA. It is estimated that dogs from 2.5% to 6.6% of any age or breed are affected by OA [1,2]. Another study suspects that 20% of dogs older than one year have OA [3]. OA causes structural and functional damage of joint structures, such as joint cartilage, ligaments, attachments of tendons, the subchondral bone, or the joint capsule. The high imbalance between anabolic and catabolic factors in the OA joint causes high production of inflammatory cytokines and higher synthesis of metalloproteinases which play an important role in the vicious circle of OA [1,4].

OA can be classified as primary osteoarthritis (POA) or secondary osteoarthritis (SOA). POA is described as OA with an idiopathic etiology. SOA is the result of factors that lead to joint dysfunction, such as congenital diseases, trauma, or metabolic disorders of the bone. There have been described various treatment options depending on the cause of the disease. As a result of different treatment options, five phenotypes have been described. These phenotypes are mentioned as post-traumatic, metabolic, aging, genetic, and pain. A post-traumatic phenotype is the result of a single or repetitive joint injury [5]. Joint dysplasia is mentioned as insufficient development of the joint, which can lead to repetitive trauma of the articular surface or periarticular soft tissue, which leads to osteoarthritis [6]. The obesity phenotype is the result of an increased load on joints due to increased force or the metabolic role of adipokines [7,8,9]. OA can develop as a result of a higher body weight load on small joint surfaces. Moreover, higher concentrations of leptin have been found in overweight dogs as another factor in OA development [10,11]. The next phenotype factor is aging. Aging was suggested as another phenotype; however, results are still not clarified due to a low number of studies. A genetic phenotype is the result of hereditary conditions. Some breeds have a predisposition to various joint diseases. Some breeds have been described as predisposed to elbow dysplasia [12,13,14]. The last phenotype is pain, which is associated with inflammation and abnormal bone remodelling in the joint [15,16]. Other factors, such as neuter status or sex, have been explored. Neutered animals were highly affected by OA due to being overweight. There were no observed differences between males and females in recent studies [17,18,19]. Results from age or neuter surveys provide some limitations, which can be influenced by different methodological approaches as well as differences between study populations [1].

Elbow joint is considered as a very common joint affected by OA with estimated 75.97% prevalence in comparison to other elbow diseases [20]. In our study, we have focused on dogs with elbow OA, which is very often a result of elbow incongruency and elbow dysplasia (ED). ED is a complex developmental skeletal disorder and can be associated with medial coronoid disease (MCD), *osteochondritis dissecans* (OCD) of the medial humeral condyle, and ununited anconeal process (UAP) which leads to cartilage erosions, osteophytes production, and subchondral bone thickening. Surgical treatment of MCD, OCD, or UAP in early stages may slow the development of OA development and prospective outcomes for patients. In animals affected by severe OA, conservative treatment is recommended [21].

The main goals in the treatment of OA are pain relief, joint-function maintenance, and deceleration of the progression of the disease. The most important aim of treatment is the retention of the functional capacity of the joint [22].

Conservative management of OA is based on nonsteroidal anti-inflammatory drugs (NSAIDs), nutritional supplements, weight restrictions, and rehabilitation exercises; however, these methods are mainly focused to relieve the clinical symptoms [23].

OA is a painful degenerative joint disease affecting the peripheral and central nervous system with nociceptive, inflammatory, and neuropathic types of pain. Due to the complex mechanism of pain, usually multimodal analgesia is necessary; however, its use can be associated with adverse effects or insufficient results. To illustrate the side-effects of NSAIDs, we have to look at recent studies, where their use was associated with side-effects such as gastrointestinal or renal toxicity as a result of COX-1 inhibition [24]. Selective COX-2 inhibitors exist as a safer alternative in OA treatment; however, their use is still the subject of research due to concerns about their side-effects in long-term use [24,25,26,27]. Another drug in OA treatment with analgesic and antipyretic abilities is paracetamol. According to the ORSI (Osteoarthritis Research Society International), paracetamol is not recommended as a single agent for OA treatment due to a lack of evidence-based studies [28,29,30,31,32,33,34]. Anti-nerve-growth-factor monoclonal antibodies, such as bedinvetmab, showed promising results in the double-blinded study, but long-term efficacy is still the subject of research [35]. Opioids such as tramadol (mu-opioid agonist, and a serotonine and noradrenaline reuptake inhibitor) were found to be useful drugs for pain treatment, but their efficacy in OA treatment is still questionable due to some studies with insufficient results. Unlike a single therapeutic agent, concurrent use of tramadol and NSAIDs showed improvement in lameness and ROM [30,33]. Gabapentin is very often used in the treatment of chronic neuropathic pain, but it does not provide the same anti-inflammatory effects as NSAIDs. The use of amantadine is still not licensed for dogs, with only one published study focusing on amantadine use in OA treatment [28].

Regenerative medicine, on the other hand, is focused to restore or replace damaged tissue and has the definite advantage of anti-inflammatory, chondrogenic, immunomodilative, antiapoptotic, and antifibrotic effects in the joint environment [36].

Multiple studies proved positive results after intra-articular MSCs application in patients with OA. Patients in these studies improved in lameness, and had pain relief, increased range of motion, and positive feedback in owners’ questionnaires [37,38,39]. Clinical application of MSCs to OA joints proved clinical improvements in human-medicine study models. However, their long-term efficacy is still questionable [40,41,42,43]. The main advantage of MSCs is their ability to secrete factors such as extracellular vesicles and bioactive molecules (chemokines, cytokines, and growth factors), which are, generally, called the secretome. MSCs offer a solution for pain relief due to a decrease in cytokines, which leads to a decrease in pain sensation [44,45]. Moreover, local application of MSCs looks more promising than systemic NSAID administration for patients with gastrointestinal, liver, or kidney diseases [33]. The crucial role of the secretome is its capability of providing immunomodulatory, anti-inflammatory, angiogenic, and anti-apoptotic qualities [46,47]. MSC are also able to promote regeneration of damaged tissue [48]. The latest clinical research showed successful autologous and allogenic MSCs application in OA treatment [44,45,49,50]. Despite the fact that MSCs showed potential as an alternative treatment for OA, allogenic MSCs administration can be associated with adverse immune response such as local skin reactions. However, minimal adverse effects were reported in canine studies [51,52,53,54]. To illustrate the MSCs pain relief as a long-term treatment option, further studies are required by the scientific community. Suppression of T-cell proliferation and M2 macrophage polarization mediated by MSCs were clarified by a description of the main effectors in MSCs-mediated secretion of PGE2, IDO, TGF-β, or TSG-6. Recent studies reflected on the potential of PGE2 to decrease the proliferation of NK cells as well as stimulate IL-10 production. Moreover, PGE2 prevents monocytes from differentiation to dendritic cells. IFN-γ, TNF-α, or IL-1β play a crucial role in MSCs’ secretion of PGE2. TSG-6 inhibits neutrophil recruitment and enhances MMPs inhibition [55,56,57,58,59,60,61,62].

The growing interest in mesenchymal stem-cell therapy in recent years by the scientific community and public opened doors for more research necessities in the information of their use, efficacy, safety, and durability. Recent studies have described the effect of MSCs in joint microenvironment as well as their long-term clinical effects [52,63].

To clarify the scientific reason for our two MSCs applications we used some published studies of short-term survival of MSCs after *in vivo* application, where the survival time of MSCs was from 7–30 days after a single injection as well as safety after repeated applications without life-threatening adverse effects [64,65,66,67,68]. To find out the beneficial effect of MSCs therapy there are successfully incorporated subjective pain scales and owner questionnaires along with objective methods such as kinematic or kinetic gait analysis which provide repeatable and quantifiable information [69].

In the process of OA, chondrocytes and other cells in synovial cells produce cytokines which are detectable in synovial fluid; namely, that are TNF-α, IL-1, IL-6, IL-2,IL-7, IL-15, and IL-21 [70,71]. It is still questionable if these cytokines directly participate in pain generation by acting on joint nociceptors [72]. However, evidence-based studies from human medicine suggest that TNF-α correlated with the pain score in a group of 47 knee OA patients who had not received prior treatment [72,73]. Small-animals studies focused on synovial fluid examination are limited due to the size of the joints and the limited amount of synovial fluid [74]. Recent MSCs applications in veterinary clinical trials on dogs proved beneficial outcomes over different time periods with various methodologies [75,76]. Mohorič et al. (2016) incorporated 22 pet dogs into a non-randomized, open, and monocentric study initially administering one cellular injection. A second injection was offered after six months to owners if the first injection did not produce expected results. Nicpoň et al. (2014) applied AD-MSCs to 12 dogs with clinical evaluation after 0, 60, 90, and 180 days of the treatment. Kriston-pál et al. applied allogenic MSCs together with 0.5% hyaluronic acid to 39 elbow joints. Significant improvement was obtained after one year follow-up [39]. Another study used umbilical cord in the treatment of knee osteoarthritis in dogs. A suspension of allogeneic UC-MSCs (1 × 10^6^ was applied intra-articulary. The follow-up examination was repeated at the 3rd, 7th, 14th, and 28th day by MRI [77].

The aim of our research was the application of current MSCs knowledge from recent literature to clinical study. We hypothesized that patients with OA have higher concentrations of inflammatory biomarkers in synovial fluid than patients without OA. The next hypothesis was that patients which received intra-articular allogenic A-MSCs injections would improve their ROM in kinematic gait analysis, due to their immunomodulatory abilities, as well as that biomarkers concentrations in their synovial fluid would decrease after therapy. The detection of synovial biomolecules might be challenging; however, they can provide an objective evaluation of the immune response to MSCs-based therapy by decreasing inflammatory cytokines in synovial fluid (SF) [67,78,79].

IL-β, TNF-α, and IL-6 are considered the most important inflammatory biomarkers in OA pathogenesis. Moreover, they are responsible for signalling pathways that activate cytokines in pathological processes. Chemokines during this process are responsible for the stimulation of cytokines, which attract inflammatory cells and lead to the worsening of pathological processes [80]. TNF-α is responsible for the synthesis of IL-6 and IL-8, as well as the induction of production of iNOS, COX-2, and PGE2 synthase [80,81,82,83]. TNF-α is a pro-inflammatory cytokine that plays a crucial role in the inflammatory response in the pathogenesis of OA. To illustrate its function, recent studies showed that it is involved in the synthesis of metalloproteinases and the stimulation of other pro-inflammatory cytokines [84,85]. IFN-γ is a cytokine that is incorporated into joint pathology; furthermore, concentrations are increased in synovial fluids in patients with OA, cartilage defects, and traumatic joint conditions [86,87,88,89,90,91]. IFN-γ is well incorporated into bone and cartilage homeostasis, mediating immune and inflammatory responses, and playing a fundamental role in the pathogenesis of joint diseases after traumatic exposure [92,93,94,95]. IL-10 is an anti-inflammatory cytokine that modulates the production of TNF-α and inhibits the release of IL-1, IL-6, IL-8, and IL-12 from monocytes/macrophages [96,97,98]. Despite the fact that IL-10 is an anti-inflammatory cytokine, increased SF levels were observed in patients with greater pain and poorer knee function [99,100,101]. Increased concentrations of IL-6 were observed in synovial fluid in obese patients, compared to patients with normal body-condition scores, which supports the theory that higher adiposity is associated with the higher concentrations in patients with end-stage OA in comparison to patients without OA [102,103,104,105]. According to other studies, concentration of IL-6 in synovial fluid is correlated with pain scales in patients with OA. Furthermore, obese patients with OA had higher concentrations of IL-6 in synovial fluid than non-obese dogs. In contrast to intact cartilage, patients with damaged cartilage had higher concentrations of IL-6 and TNF-α. GM-CSF has many functions and can act as pro-inflammatory cytokine and in dendritic cell function. Recent studies provided evidence that GM-CSF plays a key role in inflamed OA pathophysiology [22,88,102,106,107,108,109]. IL-15 raises the production of MMPs. In recent studies, the concentration of IL-15 correlated with the concentration of MMP-1 and MPP-3 in synovial fluid [99,110,111,112,113]. Monocyte chemoattractant protein (MCP-1) is secreted by synovial fibroblasts after stimulation with IL-1, TNF-α, and IFN-γ. MCP-1 is part of the metabolism of chondrocytes, osteoblasts, and synovial cells and is expressed in inflammatory conditions in synovial joints and in immune responses. Abnormally higher concentrations of MCP-1 lead to joint destruction with accelerated development of OA [114,115,116,117,118,119,120,121,122,123,124]. A recent study by Kliene et al. documented elevated synovial fluid concentrations of IL-8 and MCP-1 in the stifles of dogs with secondary OA compared with normal stifles [114].

## 2. Materials and Methods

This study was realized at the Small Animal Hospital in cooperation with the Centre for Experimental and Clinical Regenerative Medicine at the University of Veterinary Medicine and Pharmacy in Kosice and with the Associated Tissue Bank, Faculty of Medicine, P.J. Safarik University and L.Pasteur University Hospital. This study was approved by the institutional ethical committee of the University of Veterinary Medicine and Pharmacy in Kosice (EKY8/2021-02).

### 2.1. Population Study

Canine patients included in the study were dogs with mild to moderate osteoarthritis in elbow joints diagnosed after the clinical and radiographic examination. Clinical symptoms included pain during extension or flexion of elbow joints, lameness during trot or walk, and reduced range of motion (ROM). Radiographs were performed to confirm OA and joints were scored as healthy, mild, moderate, or severe according to the radiographic table in the *Canine OsteoArthritis Staging Tool* (COAST) publication and the International Elbow Working Group (IEWG) [79]. There were no criteria for breed, gender, age, or weight. Patients were also evaluated by a veterinary surgeon according to the body-condition score (BCS) described by Chun et al. [125]. In patients with bilateral OA each joint was evaluated individually. Animals with other orthopaedic or concurrent neurologic disorders were excluded from the study. Dogs that underwent surgery of the elbow (anconeal process removal/fixation, or subtotal coronoid ostectomy) were not omitted from the study, but at least two months after surgery had to pass as well as radiographic findings were needed to confirm mild or moderate OA. Patients with any kind of infections, neoplastic process, or general health problems (metabolic disorders, endocrinology disorders, gastrointestinal disorders, urinary tract diseases, or skin diseases) were not accepted for study. Patients in our study were not allowed to take any NSAIDs, corticosteroids, opioids, or attach ice-packs on joints because their use could influence the results. Patients in the treated group are summarized in Table 1. To create a control group for synovial fluid examination we used patients without clinical or radiographic signs of osteoarthritis (Table 2). Control-group patients were mainly large-breed dogs which facilitated synovial fluid collection. Synovial fluid collection from dogs in the control group was clearly for scientific reasons as well as the owners agreed to the procedure to be accomplished during sedation associated with X-ray examination or during general anaesthesia associated with castrations.

### 2.2. Isolation of MSC from Amniotic Tissue

Amniotic tissues were obtained during caesarean section (newborn puppies, n = 6, 62nd day of pregnancy) under strictly sterile conditions. Donors—American Staffordshire terrier, the weight of puppies approximately 250 g, sex ratio—4:3. Harvested amniotic sacks were then washed with PBS (Biowest, Riverside, MO, USA) containing 2% ATB + ATM (penicillin–streptomycin–amphotericin B; Sigma-Aldrich, St. Louis, MO, USA), then the tissue was cut into smaller pieces and enzymatically digested using 0.05% collagenase type IV (Thermo Fisher Scientific, Waltham, MA, USA) at 37 °C for 35 min. At the end of the incubation period, the digested tissue was filtered (through a 100 μm cell strainer) to remove non-digested tissue fragments, and the obtained fraction was centrifuged at 400× *g* for 10 min. The obtained pellet was resuspended in Dulbecco’s Modified Eagle Medium/Nutrient Mixture F-12 (DMEM-F12, Thermo Fisher Scientific, Waltham, MA, USA) + 10% Fetal Bovine Serum (FBS, Biowest, Riverside, MO, USA) + 2% ATB + ATM. Cells were plated in aT25 culture flask at a concentration of 10^6^ cells/mL and incubated in culture medium (DMEM-F12) + 10% FBS + 2% ATB + ATM at 37 °C and 5% CO_2_. Non-adherent cells were removed and the medium was subsequently changed twice a week.

### 2.3. CD Characterization of Amniotic Mesenchymal Stem Cells

When the cultivated population of A-MSCs obtained confluency of about 85–90%, cells were analyzed for mesenchymal stromal cell markers (CD29, CD44 and CD90) and for hematopoietic stem-cells markers (CD34 and CD45). Each sample was diluted to a final concentration of 2 × 10^5^ cells and centrifuged at 400× *g*/5 min. Subsequently, the supernatant was removed and the cell pellet was resuspended in 100 μL of PBS containing 3–5 μL of CD29 (MEM-101A, monoclonal antibody, phycoerythrin (PE)),CD90 (YKIX337.217, monoclonal antibody, PE), CD29 (MEM-101, monoclonal antibody, PE), CD44 (MEM-263, monoclonal antibody, PE), CD34 (1H6, monoclonal antibody, PE) and CD45 (YKIX716.13, monoclonal antibody, PE, (all Thermo Fisher, USA) and incubated for 60 min at 4 °C in the dark. At the end of the incubation period, the samples were centrifuged again at 400× *g*/5 min, the supernatant was removed and the sample was washed in 200–500 µL of washing solution (1% FBS in PBS + 0.1% Sodium Azide (Severn Biotech Ltd., Kidderminster, UK)). Cytometric analysis was performed on a BD FACS Canto^®^ flow cytometer (Becton Dickinson Biosciences, San Jose, CA, USA) equipped with a blue (488 nm) and red (633 nm) laser and six fluorescence detectors. The percentage of cells expressing individual CD traits was determined by a histogram for the respective fluorescence. The data obtained via measurement were analyzed in BD FACS Diva^TM^ analysis software. As a negative control, we used the same type of non-labeled MSC for autofluorescence control.

### 2.4. Multilineage Potential

To observe the multilineage capacity of the MSCs, we used the StemProMultilineage differential Kit (Gibco) according to the recommended protocol attached. The cells used for multilineage differentiation were from passage 3 (P3). Cells were cultured in 24-well plates with an initial density of 4 × 10^4^ cells/well for osteocytes, 8 × 10^4^ cells/well for adipocytes, and 8 × 10^4^ cells per micromass/well for chondrocytes. Each micromass was a single drop of 5 μL/6 × 10^4^ cells, which was placed in the centre of the well, and then incubated at 37 °C and 5% CO_2_ for 2 h for better adherence to the surface, and then 500 μL chondrogenic medium was added. After the recommended culture time (21 days), the cells were fixed using 4% paraformaldehyde (PFA), and the individual populations were stained with the Alizarin red staining method (Sigma) for evidence of calcium deposits in the osteoblast population—Alcian blue (Sigma) for the detection of proteoglycans in the chondroblast population and Oil red (Sigma) for the staining of fat vacuoles in the adipocyte population. A-MSCs cultured only in DMEM-F12 served as the negative control.

### 2.5. Application of MSCs

A total of two intra-articular injections were performed 14 days apart (day 0 and day 14). The first day of application was considered day 0. The application was conducted intra-articulary with a sterile needle (21 G, 0.8 mm × 40 mm). The dogs were under mild sedation of Butorfanol (0.2 mg/kg, i.v.) and Propofol (1 mg/kg, i.v.). During sedation, the infusion to all patients of NaCl 0.9% solution (with the flow of 2 mL/kg/hod) was administered intravenously. Hairs in the area of application were clipped and the skin was cleaned with Povidone-iodine soap (Betadine soap 75 mg/mL, EGIS Pharmaceuticals PLC, Budapest, Hungary), then with Skinsept MUCOS drm. sol. solution (Ecolab, Saint Paul, MN, USA). The tip of the needle was placed into the supratrochlear fossa from the medial side of the elbow. During arthrocentesis the first synovial sample was collected (average 0.3 mL) and then the volume of MSCs 0.5 mL, which represents approximately 2 × 10^6^ cells, was applied to the joint. Synovial fluid was immediately stored at −80 °C freezer after collection. During the treatment period only a short leash walk for 10 min, five times daily was recommended.

### 2.6. Kinematic Gait Analysis

**Figure 1:**
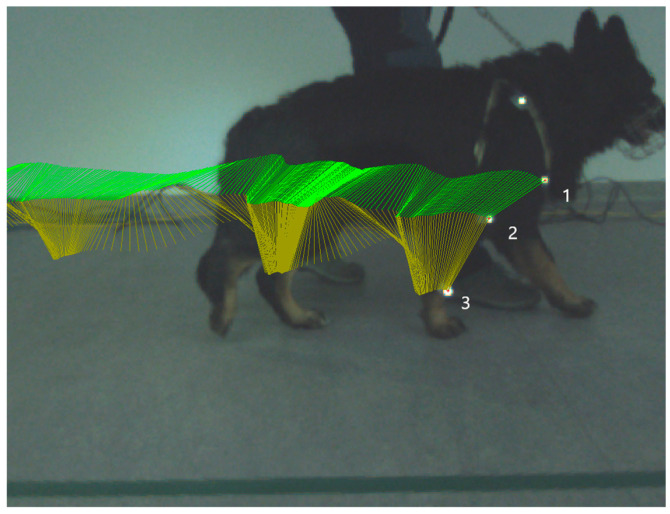
Picture illustrates kinematic gait analysis. Kinematic gait analysis was recorded and evaluated by the Simi Reality Motion System in the right thoracic limb stance phase. Each retroflective marker is placed in the specific anatomical position. Marker number 1—*tuberculum majus humeri*, marker number 2—*epicondylus lateralis humeri*, and marker number 3—*processus styloideus lateralis*. Green lines represent trajectory of connection between marker number 1 and 2, yellow lines represent trajectory of connection between marker number 2 and 3.

Kinematic gait analysis was performed by Simi Reality Motion System. Captured videos were recorded by a three-camera system (Simi Reality Motion System, Unterschleissheim, Germany and Fujifilm, Minato-ku, Tokyo, Japan) at the frequency of 120 Hz in the sagittal 2D plane on a right front limb in extension and flexion of the humeral joint and elbow joint during the stance phase and in swing phase. Three rounded retroflective markers were placed on determined anatomical landmarks (*tuberculum majus* of *humerus*, lateral humeral condyle and distal lateral aspect of styloid process). The owner on the left or right side of the dog led the dog on a leash in walk until 30 strides of each camera were obtained. All camera screens were undistorted and calibrated to obtain true values of motion. Specialized computer software was used to collect and process kinematic data using a motion-analysis program (Simi Reality Motion System, Unterschleissheim, Germany). Three markers were defined and assessed (Figure 1). After all the markers were identified, we connected specific points to create an elbow-joint angle. To be able to construct a 2D angle diagram representing elbow-joint angle, we connected points on the *tuberculum majus*, lateral humeral condyle and styloid process. Maximum flexion and extension of the elbow joint were recorded. Video recordings were analyzed using Simi Reality Motion System software. Measured values were recorded before the first mesenchymal stem-cells application (day 0) and on day 14 (before the second MSCs application) and on day 28. The range of motion (ROM) was determined by the average value of maximal extension during the stance phase minus minimal angle in flexion during the swing phase in each stride (day 0, 14, and 28).

### 2.7. Synovial Fluid Analysis

After the collection of synovial fluid samples from treated elbows (n = 13) from days 0, 14, and 28 and samples were collected from the control group (n = 9), SF samples were removed from the freezer and processed according to the prescribed protocol. Immunoassay examination by Magpix system (Luminex Corporation, Austin, TX, USA) using Milliplex map kit, Canine Cytokine Magnetic Bead Panel, 96-well plate assay (Merck KGaA, Darmstadt, Germany) was performed according to technical protocol. Magpix system was able to detect biomolecules through the fluorescent-based detection immunoassay system.

Concentrations of seven biomolecules (IFN-y, IL-6, IL-15, IL-10, MCP-1, TNF-α, and GM-CSF) were quantified, at least in duplicate, for each sample.

We sonicated the premixed bead vial for 30 s and then vortexed it for 1 min before use. Quality control 1 and Quality control 2 were reconstituted with 250 μL deionized water, mixed, and vortexed. Then, we allowed the vial to sit for 5–10min. 10× WashBuffer was diluted (60 mL of 10× WashBuffer) with 540 mL deionized water.

Preparation of Canine Cytokine Panel Standard:

We reconstituted Canine Cytokine Standard with 250 µL deionized water for analyte concentrations. The vial was inverted several times to mix. The vial was vortexed for 10 s. We allowed the vial to sit for 5–10 min. Then, we transferred the reconstituted standard to an appropriately labelled polypropylene microfuge tube. This was used as the Standard 6. To label six polypropylene microfuge tubes Standard 1 through Standard 6 we added 150 µL of Assay Buffer to each of the six tubes. Serial dilutions were prepared by adding 50 µL of the reconstituted Standard 7 to the Standard 6 tube, the well was mixed, and 50 µL was transferred from the Standard 6 to the Standard 5 tube, and then transferring 50 µL to the next tube in the series until the final standard (Standard 1) was created. The 0 pg/mL standard (Background) was Assay Buffer.

Immunoassay procedure:

1. We added 200 µL of WashBuffer into each well of the plate. The plate was sealed and mixed on a plate shaker for 10 min at room temperature (20–25 °C);
2. The WashBuffer was decanted and removed to the residual amount from all wells by inverting the plate and tapping it smartly onto absorbent towels several times;
3. 25 µL of each standard or control was added into the appropriate wells. Assay Buffer was used for 0 pg/mL standard (background);
4. 25 µL of Assay Buffer was added to the sample wells;
5. 25 µL of appropriate matrix solution was added to the background, standards, and control wells;
6. 25 μL of the sample was added into the appropriate wells;
7. The mixing bottle was vortexed and 25 μL of the premixed beads were added to each well;
8. The plate was sealed with a plate sealer. The plate was wrapped with foil and incubated with agitation on a plate shaker overnight at 4 °C or 2 h at room temperature (20–25 °C);
9. The well contents were gently removed and the plate was washed twice following instructions listed in the plate-washing section;
10. 25 µL of detection antibodies were added into each well;
11. The cover was sealed with foil and incubated with agitation on a plate shaker for 1 h at room temperature (20–25 °C);
12. 25 µL Streptavidin–Phycoerythrin was added to each well containing the 25 µL of detection antibodies;
13. The cover was sealed with foil and incubated with agitation on a plate shaker for 30 min at room temperature (20–25 °C);
14. The well contents were gently removed and the plate was washed twice following instructions listed in the plate-washing section;
15. 150 µL of Drive Fluid PLUS was added to all wells. Beads were resuspended on a plate shaker for 5 min;
16. The plate was run on MAGPIX^®^;
17. Median fluorescent intensity (MFI) data were saved and analyzed using a spline curve-fitting method for calculating cytokine/chemokines concentrations in samples.

Next, xPONENT software 4.2 for MAGPIX (Luminex Corporation, Austin, TX, USA) and Bio-Plex Manager 6.1 (BioRad Laboratories, Hercules, CA, USA) were used for data analysis. After standard curves were generated, concentrations were interpolated for each sample using a five-parameter curve-fitting equation and expressed as pg/mL.

### 2.8. Statistical Analysis

Statistical analysis of measurements from kinematic gait analysis and synovial fluid biomarkers concentration was performed by one-way ANOVA (Microsoft Office Excel, Microsoft Corporation, Redmond, WA, USA) with post-hoc Tukey’s test between each day of observation (day 0, day 14, and day 28). To analyse the correlation between ROM (day 0, day 14, and day 28) and each cytokine concentration during the treatment period (day 0, day 14, and day 28), Pearson’s correlation test and Spearman’s correlation test were used followed by the unpaired student *T*-test to detect statistical significance. To analyse the statistical significance between the control group and day 14 and day 28 of cytokine biomarkers, a Mann–Whitney U-test was performed. The results of the measurements were illustrated in Microsoft Excel Graphs and GraphPad Prism graphs (GraphPad Software Inc., La Jolla, CA, USA).

## 3. Results

### 3.1. Isolation of Canine MSCs from Amnion

**Figure 2:**
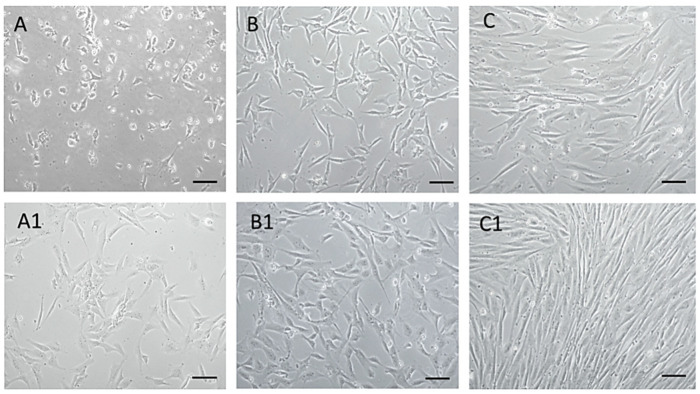
MSCs isolated from dogs’ amnion. MSCs isolated from dogs’ amnion in passage zero P0 ((**A**) DIV2, (**A1**) DIV5), first passage P1 ((**B**) DIV2, (**B1**) DIV4), and third passage P3 ((**C**) DIV4, (**C1**) DIV7). Scale bars: 50 µm.

Using the mentioned protocol, a morphologically homogeneous population of MSCs was isolated from an amnion of dogs, as can be seen from the attached photo documentation (Figure 2). At passage zero (P0) day *in vitro* 2—A (DIV2), we observed the presence of oval and bipolar cells. As the culture time increased—of A1 (DIV5), the cells changed shape and became bipolar. After reaching a confluence of 80–85% and subsequent trypsinization, cells pass from bipolar and multipolar in a fibroblast-like shape in passage P1 (B, B1). By repeated passage—P3 (C, C1) a significant homogeneity of the population, with a predominance of fibroblast-like shape cells was achieved. Thus, the cells of the isolated population showed adherence in static culture, fibroblast-like shape, and size of 40–70 μm, which mimics the properties typical for MSCs.

### 3.2. Phenotypization of MSCs

**Figure 3:**
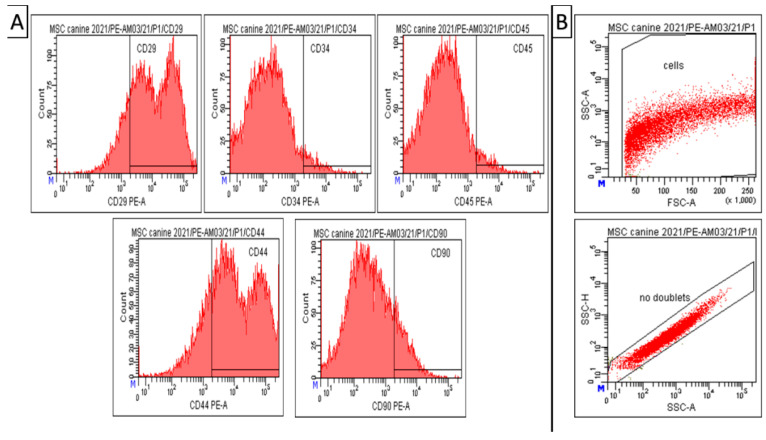
Amniotic MSCs. Results of CD analysis and gating strategy. (**A**) Canine A-MSCs from passage 1 show high positivity for CD 29 (81.5 ± 0.5%) and CD44 (77.4 ± 1.2%). However, low positivity for CD90 (14.5 ± 2.0%), CD34 (4.3± 1.0%), and CD45 (4.6 ± 0.9%). (**B**) Gating strategy—forward/sideward scatter and sidewards scatter/sideward scatter pulse height to elimination of debris and doublets. Number format explanation: 1.000 = 1000. M—Manual scaling setting. x—Multiplication.

Results of CD analyses (Figure 3) indicate that our modified isolation protocol was suitable for obtaining high homogeneity and uniformity of the mesenchymal cell population during cultivation, even in the low passage (passage 1). Canine A-MSCs from passage 1 show high positivity for CD29 (81.5 ± 0.5%) and CD44 (77.4 ± 1.2%). However, the expression of CD90 in the cell population was not significant—14.5 ± 2.0%. At the same time, the cells showed low positivity for hematopoietic markers CD34 (4.3 ± 1.0%) and CD45 (4.6 ± 0.9%), which can also be seen in the attached graph (Figure 3).Debris and doublets were eliminated by using gating strategy—forward/sideward scatter and sidewards scatter/sideward scatter pulse height (Figure 3, panel B). The viability of observed cells varied between 85–93%.

### 3.3. Multilineage Potential

**Figure 4:**
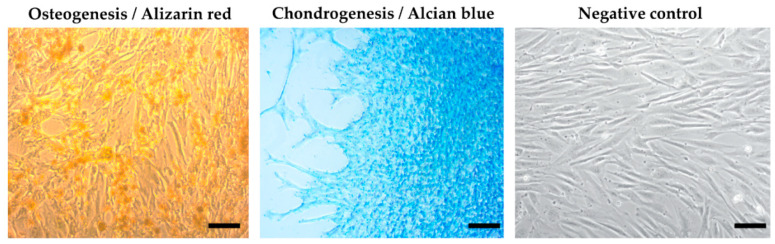
Results of multilineage differentiation.Multilineage potential of canine MSCs from amnion. Canine A-MSCs show a high capability to differentiate in osteoblast (presence of calcium depositsdetected by Alizarin red) and chondrocytes (presence of glycoproteoglycans detected by Alcian blue) after culturing in StemProMultilineage differential kit. Scale bars: 50 µm.

Using a multilineage differentiation kit and the essential supplements (Gibco), the recommended culture protocol, and staining methods, we confirmed the ability of MSCs isolated from an amnion of dogs to differentiate into osteocytes and chondrocytes. The ability to differentiate into an adipogenic line was absent (Figure 4).

### 3.4. Clinical Examination Post MSCs Application

A-MSCs application showed no adverse effects on the patients’ general health. Rectal temperature was in reference value for all patients. No swelling or redness in the area of application was detected. Transient lameness was observed in the first 12–24 h post application in two patients; however, this lameness resolved without recurrence within 24 h in both patients. Recovery from sedation was fast, without vocalizing, inappetence, or problems with urination.

### 3.5. Kinematic Gait Analysis

**Figure 5:**
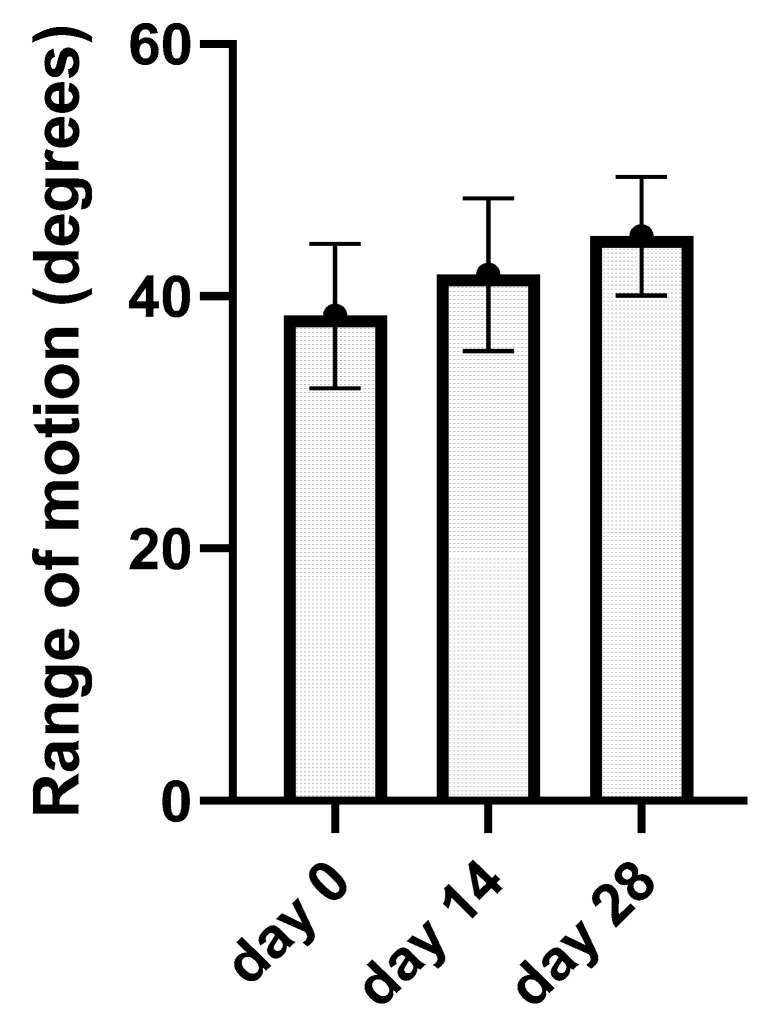
The bar graph represents kinematic gait analysis results. The average range of motion in degrees for a single day of application (day 0, day 14, and day 28) with standard deviation are summarized.

Kinematic gait analysis was successfully performed on all 13 elbows from the treated group on day 0, day 14, and day 28. After kinematic data processing, average ROM data were revealed for day 0 (38.45 ± 5.74°), day 14 (41.7 ± 6.04°), and day 28 (44.78 ± 4.69°). Average ROM values and standard deviation from each joint were incorporated into the bar graph (Figure 5). This graph has showed the average values of ROM after every application. Using one-way ANOVA test with the post hoc Tukey test, we obtain *p*-value for each elbow during the treatment period. Results have been statistically significant (*p* < 0.05) in nine joints after treatment (day 28) and in four elbows not statistically significant (*p >* 0.05); these results are summarized in the graph (Table 3). ROM analysis between day 0 and day 14 as well as between day 14 and day 28 was not statistically significant.

### 3.6. Synovial Fluid Analysis

**Figure 6:**
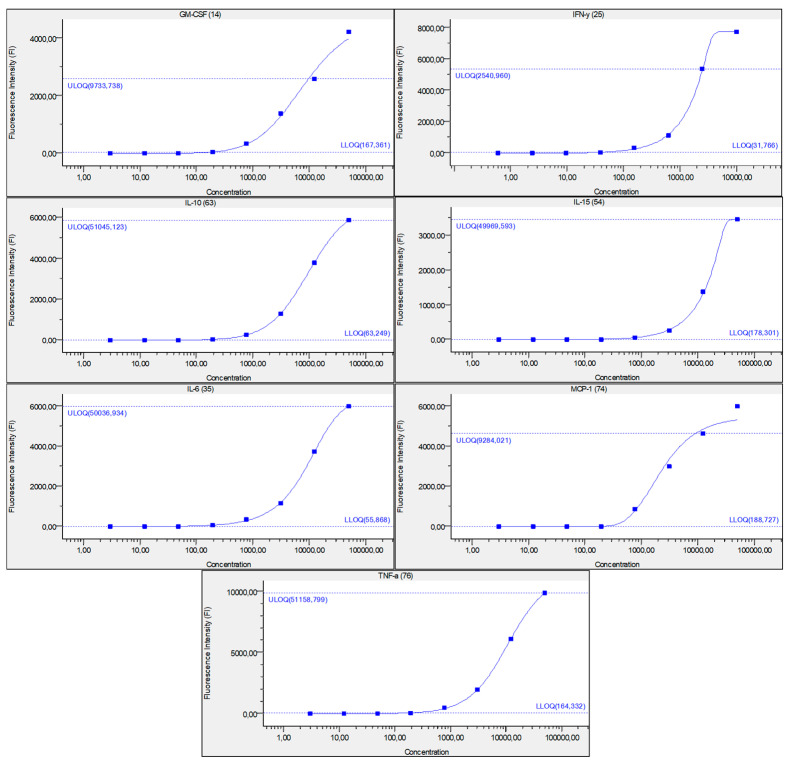
Figure represents standard curve for IFN-γ, IL-6, IL-15, IL-10, MCP-1, TNF-α, and GM-CSF, after serially diluted recombinant cytokine standards. *Y*-axis describes fluorescence intensity (FI) and *X*-axis describes concentration (pg/mL). Numbers in brackets are the Luminex beads numbers that were used. Number format explanation: 1,0 = 1.

**Figure 7:**
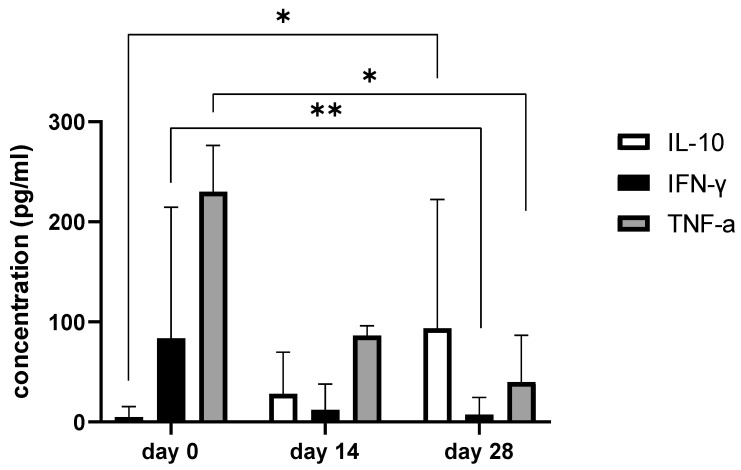
The bar graph shows concentration changes of IL-10, IFN-γ, and TNF-α during day 0, day 14, and day 28. Note statistical significance *p*< 0.05 (*) for IL-10 and TNF-α concentrations between day 0 and day 28 and statistical significance at level 0.001 (**) in IFN-γ concentrations between day 0 and day 28.

**Figure 8:**
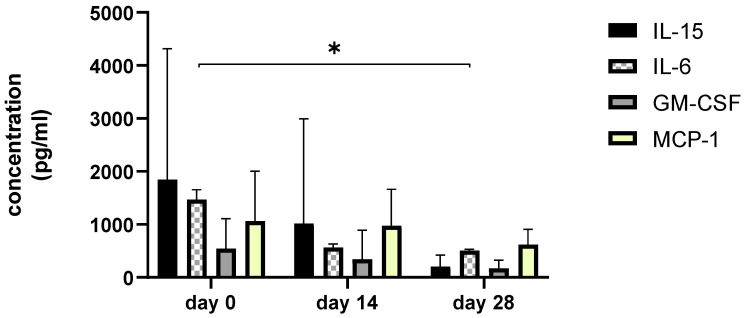
The bar graph shows concentration changes of level IL-15, IL-6, GM-CSF, and MCP-1 during day 0, day 14, and day 28. Asterisks represent statistical significance *p* < 0.05 for IL-6 concentration between day 0 and day 28.

**Figure 9:**
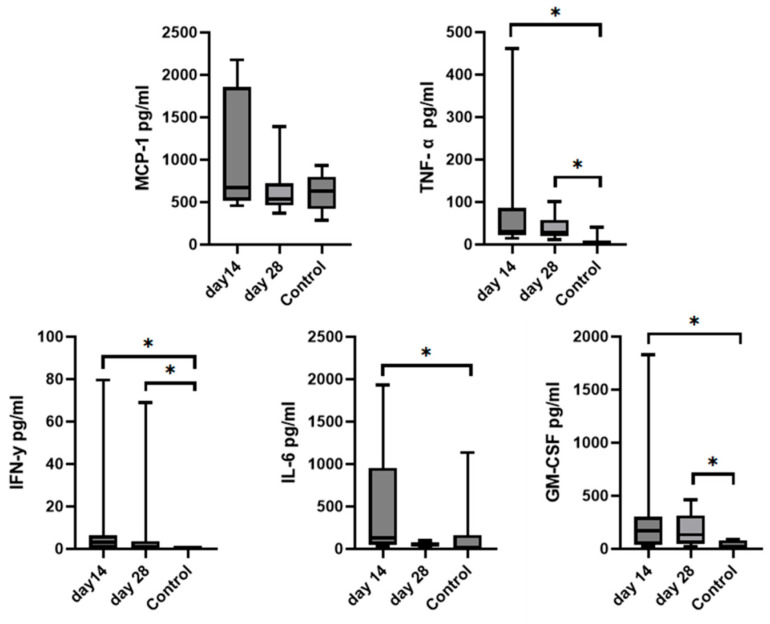
Graphs represent comparison of mean biomarkers concentration with SD of TNF-α, MCP-1, IL-6, IFN-γ, and GM-CSF in SF between the control group and the treated group (day 14 and day 28) after MSCs treatment. IL-10 and IL-15 concentrations were not able to be measured in the control group due to low concentrations. Asterisks represent statistical significance *p*< 0.05.

**Figure 10:**
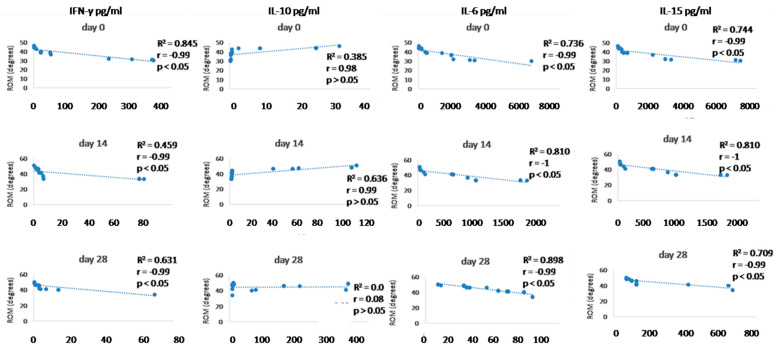
Correlations between cytokines (IFN- γ, IL-10, IL-6, and IL-15) concentrations (day 0, day 14, and day 28) and ROM (day 0, day 14, and day 28). R^2^ represents the square of the Pearson’s correlation coefficient, r represents Spearman’s correlation coefficient. *p* < 0.05 represents correlation which is statistically significant, *p* > 0.05 means correlation is not statistically significant.

**Figure 11:**
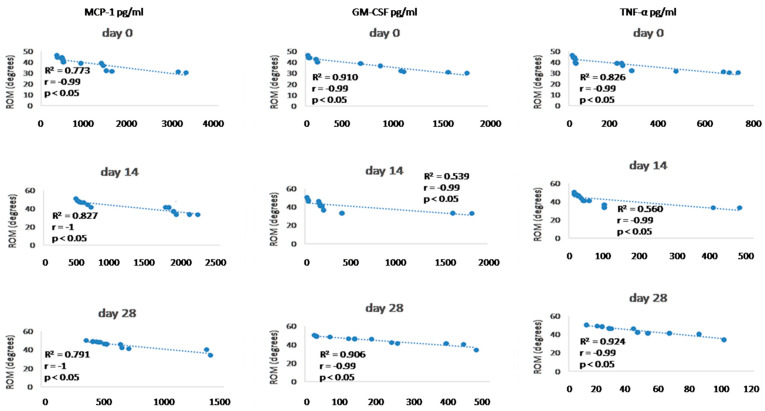
Correlations between cytokines (MCP-1, GM-CSF, and TNF- α) concentrations (day 0, day 14, and day 28) and ROM (day 0, day 14, and day 28). R^2^ represents the square of the Pearson’s correlation coefficient, r represents Spearman’s correlation coefficient. *p* < 0.05 represents correlation which is statistically significant.

Since we finished the prescribed protocol of Milliplex map kit, Canine Cytokine Magnetic Bead Panel, 96-well plate assay (Merck KGaA, Darmstadt, Germany) as well as created the standard curve (Figure 6), we have observed measured concentrations (pg/mL) of IFN-γ, IL-6, IL-15, IL-10, MCP-1, TNF-α, and GM-CSF for the treated group and the control group (Table 4 and Table 5). In the control group, due to low concentrations, the IL-10 and IL- 15 were not detected (OOR<). For every biomarker we detected decrement at day 14 and day 28 in comparison to day 0, except IL-10 measured concentrations were higher at days 14 and 28 in comparison to day 0 (Figure 7). Statistical significance (*p*< 0.05) was observed in IFN-γ, IL-6, IL-10, and TNF-α between days 0 and day 28 (Figure 7 and Figure 8). Mann–Whitney U-test showed statistical significance (*p* < 0.05) in TNF-α, IFN-γ, and GM-CSF concentrations between day 14 and the control group as well as at day 28 and the control group. IL-6 concentrations showed statistical significance (*p* < 0.05) between day 14 and the control group. MCP-1 concentration showed no statistical significance with the control group (Figure 9). IL-10 showed a positive correlation with ROM at day 0, day 14, and a mild positive correlation at day 28. IFN-γ, IL-6, and IL-15 showed a negative correlation with ROM at day 0, day 14, and day 28. *p* < 0.05 was statistically significant in the correlation of ROM and IFN-γ, IL-6, and IL-15 at day 0, day 14, and day 28 (Figure 10). MCP-1, GM-CSF, and TNF- α showed negative correlation with ROM at day 0, day 14, and day 28. Correlations’ statistical significance (*p* < 0.05) in MCP-1, GM-CSF, and TNF-α and ROM at day 0, day 14, and day 28 was observed (Figure 11).

## 4. Discussion

Elbow osteoarthritis is a very common source of lameness in dogs and is usually a consequence of elbow dysplasia. In many patients with mild OA secondary to ED are beneficial surgical procedures to improve the clinical status of the patient [126,127]. In some cases, surgical treatment cannot bring the expected benefits because of high-grade OA (severe) or the owner’s rejection to undergo a surgical procedure for their dog. Surgical treatments of elbow diseases can be classified as a symptom-oriented treatments or disease-modifying treatments which aim to correct the suspected cause of the disease and modulate the disease process [128]. Results of surgical treatment depend on several factors, such as the severity of pre-existing OA, the age of the patient, the patient’s level of activity, and the modified Outerbridge score [23,128,129]. The best prognosis is expected in young animals with a low grade of OA and post-operative rehabilitation. Due to the lack of objective data on a specific surgical treatment, it is hard to predict individual responses without objective measurements of outcomes, but surgical therapy might offer improved outcomes compared to conventional medical management [23,130].

OA can be the result of hereditary conditions. Some breeds have a predisposition to various joint diseases due to a predisposition related to breed standards and genetic/heritability components. Some breeds have been described as predisposed to elbow dysplasia, such as Mastiffs, Boxers, Italian Corso dogs, German shepherds, and Golden and Labrador retrievers [12,13,14]. The state of OA was identified as mild (seven elbows) and moderate (six elbows), and elbows were evaluated according to COAST and IEWG [79,131].

To understand the risk factors affecting MSCs *in vivo*, we have to look at recent studies, because forces applied to the cells can affect the cytoskeleton, adhesion to the extracellular matrix, and intracellular proteins. This mechanism of action can negatively influence cell differentiation. It is important to stress that mechanical microenvironment affect the survival of MSCs and their regenerative abilities [65,75,132,133]. This is why we ordered the owners to keep the dog in restricted motion during MSCs application, since repeated stress on a joint could influence the study [65,75,133]. According to this information, we assume that the survival time of injected MSCs in patients with moderate OA could be shorter in comparison to patients with mild OA. On the other hand, a higher grade of OA is associated with higher concentrations of inflammatory cytokines in the joint. The release of cytokines such as IL-1, IL-4, IL-9,IL-13, and TNF-α and enzymes such as ADAMTs and MMPs, which are also produced by chondrocytes, osteoblasts, and synoviocytes, enhance the anti-inflammatory properties of MSCs [134,135,136]. Studies investigating the mechanism of MSCs action identified that IFN-γ, TNF-α, IL-1β, and IL-17 are responsible for the induction of MSCs into an anti-inflammatory state. A high level of inflammation in the environment will cause a higher production of anti-inflammatory and immunomodulatory factors by MSCs. These factors are mainly TSG-6, IL-6, and PGE2. This means that the immunomodulatory effect of MSCs depends on the severity of tissue inflammation [137,138,139,140,141]. Another factor that can influence the study are NSAIDs, which were described as inhibitors of MSCs *in vitro* [142] and *in vivo* [143]. NSAIDs inhibit proliferation, differentiation, and migration of MSCs [144,145]. The cold environment was also described as an inhibitor of MSCs [146,147]. Due to this information, ice-packs and NSAIDs were omitted from our study. The use of opioids was also prohibited due to their potential influence on kinematic gait analysis.

In animal research, other factors are also mentioned that may influence OA pathogenesis. In older patients, it was described as a process of higher cytokine production in older chondrocytes as a result of the accumulation of advanced glycation end products in chondrocytes [56,148,149]. The average age of dogs in the treated group was 5.4 years, and in the control group, the average age was 3.1 years, so we can assume that age was one factor influencing the stage of OA and the concentration of synovial fluid biomarkers.

Adipokines produced by adipose tissue were considered another factor in OA pathology in obese patients. In our study, we were not focused on the weight of the patient, but we evaluated dogs by body-condition score (BCS). BCS may help to decide if the patient suffers from obesity [56,148,149]. In our study, we had two dogs with grade 5 BCS; German shepherd evaluation of ROM was not statistically significant between day 0 and day 28. The second patient was a Golden retriever with bilateral osteoarthritis, however, in this patient, both elbows were statistically significant in ROM examination. We can suspect that a higher BCS score should influence biomarker concentrations.

Mesenchymal stem cells bring additional advantages to inflamed joints [150]. MSCs have abilities to restore damaged cartilage to its previous state [151], immunomodulatory effects in rheumatic arthritis treatment [152], anti-inflammatory effects in OA joints in equine, canine, and rodent studies [153,154], moreover, an anti-apoptotic and anti-fibrotic effect [155]. Osteoarthritis is associated also with cartilage degradation, softening, swelling, fissuring, fibrillation, or eburnation [156]. Despite the fact that MSCs are able to promote cartilage regeneration [157], *in vivo* efficacy is still not sufficient for clinical use [158,159].

The most common form of MSCs administration in multiple studies was by intra-articular application to dogs suffering from OA, however, some studies used systemic (intravenous) or acupuncture application due to MSCs’ ability to migrate to lesion sites. To confirm that migration theory, more studies need to be done; nevertheless, intravenous application should be more accessible for veterinary practices. Some studies preferred the usage of scaffold-containing MSCs; moreover, these studies had better results in comparison to the intra-articular application [8,9,14,15,16,160]. In our study, we focused on repeated intra-articular application with a dose of approximately 2 × 10^6^ cells. In contrast to other studies where variation between doses was from 1 × 10^6^ to > 15 × 10^6^ cells for AD-MSCs single intra-articular application [44,61,161,162,163,164,165,166,167,168,169,170,171] or 5 × 10^7^ MSCs were seeded on a scaffold and 1 × 10^6^ to 1 × 10^7^ MSCs were injected IA (up to four injections) [77,160,162]. Unlike studies where only cell-adhesion and cell-surface markers were detected, we have performed characterization of MSCs before clinical use [77,160]. The efficacy of MSCs treatment was evaluated by diverse methods, such as orthopaedic evaluation or owner questioners, and kinetic or kinematic gait analysis [37,39,52,171,172,173,174,175]. Beneficial results were obtained in studies that assessed lameness, pain, or ROM after AD-MSCs administration [37,39,58,172,173] as well as BM-MSCs. Good results were observed in human studies describing clinical outcomes after BM-MSCs application [37,172,173,176,177]. Some studies assessed the cartilage healing by arthroscopic or MRI examination after intra-articular application [39,77], however, these methods were omitted in our study due to financial issues. Other studies performed biomechanical analyses of cartilage and subchondral bone [160,161]. Quite a lot of studies were aimed at synovial fluid biomarker evaluation, with the detection of a decrease in chronic inflammation after MSCs application as well as a decrease in cytokines such as TNF-α, IFN-γ in the joint tissue and synovial fluid; moreover, some studies discovered the effect of the intra-articular application on inflammatory markers in the peripheral blood [160]. Our aim was to utilize recent MSCs-based research in our clinical study. The source of MSCs were amniotic membranes from dogs. A-MSCs were successfully implemented in various studies [178,179,180]. Many studies described the positive efficacy of MSC after intra-articular application in OA, but, mostly, the sources were adipose tissue (perivascular fraction) or bone marrow [181,182,183,184]. Studies with amniotic MSCs application in canine patients are limited, however, their isolation, characterization, differentiation, and safety are well described [185,186]. Recent studies provided immunomodulatory abilities in OA synovial fluids after A-MSCs exposure [187,188].

MSCs therapy might be associated with some adverse effects [188]; our clinical study design, however, has not shown unfordable effects on patients’ health status. Serious negative side-effects were not reported in modeled-based studies [52,171,172,173,174,175,189]. One dog was presented with lameness after MSCs application due to repeated attempts at inserting the needle into the joint in a study of 10 dogs with hip OA [168], another dog showed deterioration in lameness after AD-MSCs, and two of the dogs experienced skin allergy [54]. In a survey focused on AD-MSCs, two dogs had joint swelling for several days [39]. Some studies reported transient mild inflammatory responses in dogs following MSC administration [52,190].

In our protocol, three passages (P3) of A-MSCs were made, and every passage was characterized due to the properties of A-MSCs which could vary with each passage [177]. Cultivated A-MSCs fulfilled the criteria for MSCs according to the International Society for Cellular Therapy, as described in the previous studies [191,192]. Based on that, we have validated using flow-cytometry analysis, where A-MSCs provided a high expression of CD29 (81.5%) and CD44 (77.4%), and a low expression for CD34 (4.3%) and CD45 (4.6%). Moreover, we confirmed the multilineage potential of A-MSCs, which demonstrated the ability to differentiate into osteocytes and chondrocytes under recommended conditions. 

Additionally, studies indicate a low survival rate of MSCs *in vivo* [76,193]. Moreover, repetitive delivery of A-MSCs can keep viable MSC cells at the injury site for a longer period [65,66]. Due to this reason, we have decided for repeated A-MSCs application. In contrast, another study has described that MSCs survived in a lesion 30 days after application [194]. To understand the risk factors affecting MSCs *in vivo* we have to take a closer look at recent studies because forces applied to the cells can negatively influence the cytoskeleton, adhesion to the extracellular matrix, and intracellular proteins. This mechanism of action can negatively influence cell differentiation. Besides that, the mechanical microenvironment affects the survival of MSCs and their regenerative abilities [75,133,195].

To analyse efficacy of the A-MSC therapy, objective gait analysis was performed. Kinematic gait analysis provides a modern procedure that is able to detect the patients’ range of motion during the movement in 2D or 3D [196,197]. However, kinematic gait analysis along with kinetic gait analysis can provide a more sufficient evaluation of therapeutic modalities [69]. Goniometry is a feasible technique for many clinicians, but cannot obtain locomotion values during a walk, trot, or run. Breed-specific kinematic gait analyses were described due to different anatomic constitutions which can affect a range of motion values [196]. In recent studies were values of maximal elbow extension 154.28 ± 9.64 degrees in Labradors and the minimum angle in flexion was 90.52 ± 11.16. In Rottweilers, maximum extension was 148.85 ± 9.15 and 93.99 ± 10.19 degrees in flexion [196]. Additionally, a study on German shepherds detected maximal elbow extension 131.77 degrees and 68.15 degrees in flexion [198]. Moreover, kinematic gait analysis in beagles proved 138.1 ± 11.1 degrees in maximal extension and 73.2 ± 9.0 degrees in flexion. Another study detected 151.86 degrees in maximal extension and 91.03 degrees in flexion in American pit bull terriers [199,200]. These studies were not focused on ROM expression (maximal extension angle minus minimal flexion angle) but mathematically it is possible to calculate average ROM measured values. Our study detected values for ROM in the walk for a mixed group of dogs at day 0 (38.45 ± 5.74°), day 14 (41.7 ± 6.04°), and day 28 (44.78 ± 4.69°). In kinematic studies of large-breed dogs were average ROM values for elbow joints 48.1° and 52.9° in walking [201,202]. According to the previous studies as well as our findings, we assume that our kinematic gait analysis proved that application of A-MSCs improved the patient gait ROM, however, results have proved that in nine joints after treatment (day 28) were statistically significant (*p* < 0.05) and four elbows not statistically significant improvement (*p >* 0.05). IL-10 showed positive correlation with ROM at day 0, day 14, and mild positive correlation at day 28. IFN-γ, IL-6, IL -15 showed negative correlation with ROM at day 0, day 14, and day 28. *p* < 0.05 was statistically significant in correlation of ROM and IFN-γ, IL-6, and IL-15 at day 0, day 14, and day 28 (Figure 10). MCP-1, GM-CSF, and TNF-α showed a negative correlation with ROM at day 0, day 14, and day 28. Statistical significance (*p* < 0.05) in MCP-1, GM-CSF, and TNF-α and ROM correlation at day 0, day 14, and day 28 was observed (Figure 11). According to recent studies was positive correlation detected in comparison to osteoarthritis scores, where higher scores described worse clinical outcome and higher state of OA [73,86].

Furthermore, in our study we have also focused on changes in SF biomarkers such as IFN-γ, IL-6, IL-15, IL-10, MCP-1, TNF-α, and GM-CSF before and after A-MSCs application as well as on the SF concentration in the control group. Interesting results were obtained from research focused on different animal OA models [74]. Although it was mentioned in this review that synovial fluid biomarkers represent the joint environment better than biomarkers from urine and serum, there are currently no recommendations for which biomarker should be examined. Despite the fact that predominant pro-inflammatory cytokines such as IL-1β, TNF-α, IL-15, or IL-6 are well described in human medicine research [72,203], it is not documented which biomarker is the gold standard for evaluating osteoarthritis in animal species. Legrand et al. summarized data from 69 animal studies focused on OA biomarkers. A wide range of animal models have been used in OA biomarker detection [204,205,206]. A combination of different biomarkers can provide beneficial information regarding the progression of degenerative joint disease as well as information about catabolism and anabolism processes [74,206,207]. The values of these biomarkers were measured in synovial fluid by multiple studies [208,209,210,211]. Cytokines are cell signalling proteins that mediate many physiological processes. Secretion of pro-inflammatory cytokines leads to inflammatory responses accomplished by macrophages, T–cells and B-cells [212]. Osteoarthritis is mentioned as an inflammatory process associated with active secretion of TNF-α and IL-1β [213,214]. IL-6 and TNF-α are released by synovial fibroblasts and chondrocytes leading to inflammation and cartilage breakdown [42]. IFN-γ or IL-10 correlated with radiographic OA severity or joint pain [86]. IL-15 was described as a cytokine responsible for cartilage degradation, GM-CSF recently showed importance as an OA pain mediator and MCP-1 has been recognized in higher SF concentration in OA joints compared to control groups [215,216,217]. The concentration of these biomarkers may be influenced by OA state of the joint, which includes the pathogenesis process in cartilage, synovial membrane, or ligaments [218]. Elevation of TNF-α may be associated with cartilage defects or osteochondral healing [219]. Additionally, TNF-α is up-regulated in collagen and aggrecan cartilage destruction in joints affected by rheumatoid arthritis and is responsible for necroptosis in injured and inflamed tissue [220]. Some animal studies showed decreasing of TNF-α in synovial fluid after repeated MSCs application [221] as well as in other tissues [222,223,224]. IFN-γ is a product of T-cells and NK-cells and its concentrations in synovial fluid are higher in patients with severe OA and cartilage defects [90]. However, chondrocytes treatment with IFN-y increased transcription of TNF-α, IL-6, MMP-13, and PKR and decreased IL-1β expression with down-regulation of MMP-1 and MMP-3 [225,226], IFN-γ-induced apoptosis of cells, and inflammatory response [227,228]. Moreover, IFN-y improved and enhanced the activity of MSCs [81]. IL-6 is described as a pro-inflammatory cytokine and its concentrations in OA joints correlated with MMP-3 [229,230]. Furthermore, IL-6 is mentioned as a helpful marker for OA detection [230]. Higher concentrations of IL-6 were detected in patients with cartilage defects as well as in joints with cartilage healing [88]. Besides that, SF in OA joints up-regulated the expression of IL-6 in MSCs [231]. On the other hand, some studies reported a decrease in IL-6 after MSCs–conditioned medium exposition [67,232]. Moreover, MSCs decreased serum and synovial fluid levels of IL-6 [50,217]. IL-10 appears as an important part of cartilage immunoregulation and homeostasis [233]. To illustrate MSCs’ relation to IL-10, the previous studies proved that MSCs express IL-10 which inhibits arthritis models [234]. Intra-articular application in OA joints increased IL-10 concentration [235]. Increased values of IL-15 were detected in SF in early OA stages, which correlated with IL-6 concentration [99]. GM-CSF and MCP-1 were also detected in serum and synovial fluids in patients with osteoarthritis [227,236,237]. We managed to evaluate cytokines by multiplex fluorescent micro-bead immunoassay (FMIA) designed to detect antibodies in synovial fluid samples. The previous studies described that FMIA is more sensitive than many commercial ELISA kits [238,239,240]. In our study, we were able to detect concentrations of TNF-α 0.836 (0.09–4.11) pg/mL, IL-6 17.50 (1.04–113.38) pg/mL, IFN-y 0.31 (0.07–1.05) pg/mL, GM-CSF 33,12 (1.14–92.10) pg/mL, and MCP-1 60.505 (33.50–93.0) pg/mL in the control group of patients (n = 9) without radiographic and clinical signs of OA. Concentrations of IL-10 and IL-15 were not detected with fluorescence immunoassay due to low concentrations (OOR<). Many studies which detected inflammatory synovial biomarkers were published in recent years, but different detection methods and a lack of control groups cannot clearly clarify reference values [86,208]. In comparison to the control group, on the 14th and 28th day of MSCs application, we detected statistical significance in some cytokines. However, IL-6 concentration was higher in some joints compared to day 28. Our study showed that after repeated A-MSC application decreased concentration of inflammatory markers was reached as well as IL-10 concentration increased at day 28. Statistical significance *p* < 0.05 was obtained in IL-6, IL-10, and TNF-α, and statistical significance of *p* level at 0.001 at IFN- y between day 0 and day 28. Clarification of the mechanism of action of MSCs in inflamed joints is still the subject of research [240]. However, their main ability attenuate the OA joints is through the production of cytokines and growth factors [241,242,243]. To illustrate MSCs’ mechanism of action we have to look closer into published studies which described MSCs cell-to-cell interaction and their stimulation by inflammatory cytokines such as IFN-y, TNF-α, IL-1, or IL-1β in the environment. Additionally, MSCs are also able to avoid T-cell proliferation and ensure macrophage polarization [244,245,246]. Similarly to our study, other researchers described the up-regulation of IL-10 and down-regulation of IL-1β, IL-6, and TNF-α in OA joint by MSCs exposition [247,248]. Nevertheless, the efficacy of cell-based treatment is affected by many factors including joint state and etiology of the degenerative process. The repeated application brings satisfactory results, however, the duration of efficacy is still questionable and subject to further research [249,250]. The placebo group is beneficial for interpreting results [251], however, we could not afford one due to a limited number of patients. Moreover, the owners refused to participate in a placebo trial study.

## 5. Conclusions

Our study proved favourable efficacy for patient movement (ROM) and a decrease in inflammatory cytokines after repeated A-MSCs application without adverse effects on the patients’ health. First, our study was established to obtain and prepare processed and characterized A-MSCs for cell-based therapy. Furthermore, we have created a control group of dogs to evaluate inflammatory biomarkers in SF in comparison to a treated group of patients at day 0, day 14, and day 28. Despite the fact that our treated and control group contained only a small number of patients, the study offers options and feasible methods as a stepping stone for other A-MSCs-based therapies in canine models. Unlike bone marrow-derived MSCs or adipose tissue-derived MSCs, the lack of A-MSCs clinical studies based on their application to canine patients can be a consequence of the inappropriate awareness of clinicians. A-MSCs are easily obtainable from c-sections and offer low-risk morbidity for donors.

## Figures and Tables

**Table 1:** The table shows patients included in the therapy group (8 dogs, 13 elbow joints included in the study). Abbreviation: GS—German shepherd, CB—crossbreed, LR—Labrador retriever, APT—American pit bull terrier, CS—cocker spaniel, GS—giant schnauzer. SE—single elbow, BE—bilateral elbow. SCO—subtotal coronoid ostectomy, APR—anconeal process removal. COAST—canine osteoarthritis staging tool. COAST—canine osteoarthritis staging tool, BCS—body condition score.

	Breed	Gender	Age (Years)	COAST Rad. Assessment, IEWG	Number of Joints	Previous Surgical Procedures	Pain upon Manipulation	BCS/Weight (kg)
1	GS	Male	5	Moderate	SE	SCO	Mild	5/40 kg
2	CB	Male	6	Moderate	BE	SCO	Mild	3/21 kg
3	LR	Male	2	Mild	BE	SCO	None	3/36 kg
4	APT	Female	7	Moderate	SE	None	Mild	3/25 kg
5	LR	Male	3	Mild	BE	SCO	None	5/40.3 kg
6	CS	Male	8	Moderate	BE	SCO	Mild	3/16.4 kg
7	APT	Female	8	Mild	BE	None	Mild	3/22.5 kg
8	GS	Male	4	Mild	SE	APR	None	3/36 kg

**Table 2:** The table shows patients which were included in the control group. Abbreviation: GS—German shepherd, CAS—central Asian shepherd dog, CS—Caucasian shepherd, LR—Labrador retriever, APT—American pit bull terrier, CS—Slovak cuvac, D—Doberman. NRSOO—No radiographic signs of osteoarthritis. COAST—canine osteoarthritis staging tool.

	Breed	Gender	Age (Years)	Type of Joint	COAST Rad. Assessment	Pain Upon Manipulation
1	GS	male	2	elbow	NRSOO	None
2	CAS	male	2	elbow	NRSOO	None
3	GS	female	5	elbow	NRSOO	None
4	CS	male	2	elbow	NRSOO	None
5	GS	male	5	elbow	NRSOO	None
6	LR	female	5	elbow	NRSOO	None
7	APT	male	2	elbow	NRSOO	None
8	SC	male	2	elbow	NRSOO	None
9	D	female	3	elbow	NRSOO	None

**Table 3:** *p*—value of kinematic gait analysis between day 0 and day 28, graph in 13 elbows after MSCs application. Abbreviations: RE—right elbow, LE—left elbow, ss—statistical significance (*p* < 0.05), ns—not statistically significant (*p* > 0.05).

Patient	Statistical Significance in ROM
German shepherd	*p* value = 0.1300 ns
Crossbreed, RE	*p* value = 0.0013 ss
Crossbreed, LE	*p* value = 0.0011 ss
Labrador retriever, RE	*p* value = 0.0130 ss
Labrador retriever, LE	*p* value = 0.0110 ss
American pit bull terrier	*p* value = 0.0900 ns
Labrador retriever, RE	*p* value = 0.0006 ss
Labrador retriever, LE	*p* value = 0.0112 ss
Cocker spaniel, RE	*p* value = 0.3600 ns
Cocker spaniel, LE	*p* value = 0.0010 ss
American pit bull terrier, RE	*p* value = 0.0002 ss
American pit bull terrier, LE	*p* value = 0.0020 ss
German shepherd	*p* value = 0.4100 ns

**Table 4:** Mean concentration levels of biomarkers in SF with a range of concentrations in brackets during day 0, day 14, and day 28. *p* value at level 0.001 (**), at level *p* < 0.05 (*). ns—not statistical significance (*p* > 0.05).

Biomarker in SF	Day 0. Mean (Range)	Day14. Mean (Range)	Day28. Mean (Range)	*p*-Value	Statistical Significance
IFN-y (pg/mL)	83.58 (4.7–345.7)	12.1 (1.6–79.5)	7.40 (1.1–68.9)	0.001	**
IL-10 (pg/mL)	4.9 (2.6–31.28)	28.1 (12.3–109.6)	93.64 (8.63–342.8)	0.009	*
IL-15 (pg/mL)	1848.2 (201.1–7031.1)	1018.2 (8.6–5674.5)	206.6 (72.61–665.4)	0.124	ns
IL-6 (pg/mL)	1468.7 (41.0–6409.2)	565.2 (35.9–1930.2)	50.7 (11.4–96.2)	0.011	*
MCP-1 (pg/mL)	1066.2 (453.1–3271)	977.5(452.1–2176.5)	619.9 (369.2–1350.3)	0.343	ns
TNF-α (pg/mL)	230.3 (14.8–668.4)	86.3 (13.2–461.7)	39.1 (11.3–100.6)	0.012	*
GM-CSF (pg/mL)	549.1 (36.7–1567)	344.4 (22.7–1828.3)	172.1 (25.8–381.1)	0.163	ns

**Table 5:** The mean concentration of biomarkers in SF with a range of concentrations in brackets was measured from the control group. IL-10 and IL-15 were not able to be measured due to low concentrations in samples (OOR<—out of range below).

Mediator in SF	Mean (Range)
IFN-y (pg/mL)	3.1 (0.7–10.5)
IL-10 (pg/mL)	OOR<
IL-15 (pg/mL)	OOR<
IL-6 (pg/mL)	17.50 (1.04–113.38)
MCP-1 (pg/mL)	60.50 (33.50–93.0)
TNF-α (pg/mL)	0.83 (0.09–4.11)
GM-CSF (pg/mL)	33.12 (1.14–92.10)

## Data Availability

The data presented in this study are available on request from the corresponding author.
